# ICTV Virus Taxonomy Profile: *Belpaoviridae* 2021

**DOI:** 10.1099/jgv.0.001688

**Published:** 2021-11-11

**Authors:** Beatriz Soriano, Mart Krupovic, Carlos Llorens, ICTV Report Consortium

**Affiliations:** ^1^​ Biotechvana, Scientific Park University of Valencia, 46980, Paterna, Valencia, Spain; ^2^​ Archaeal Virology Unit, Institut Pasteur, Université de Paris, F-75015 Paris, France

**Keywords:** ICTV Profile, taxonomy, *Belpaoviridae*, *Semotivirus*

## Abstract

The family *Belpaoviridae* comprises metazoan-infecting reverse-transcribing viruses with long terminal repeats, commonly known as Bel/Pao LTR retrotransposons. These viruses share evolutionary history and genes involved in genome replication and virion formation with reverse-transcribing viruses of the families *Metaviridae*, *Pseudoviridae*, *Retroviridae* and *Caulimoviridae*. These five families form the order *Ortervirales*. This is a summary of the ICTV Report on the family *Belpaoviridae*, which is available at ictv.global/report/belpaoviridae.

## Virion

Little is known about the virion morphology of belpaovirids. However, given that belpaovirids encode a Gag polyprotein with nucleocapsid and capsid protein domains homologous to those of other members of the order *Ortervirales* [1, 2], their replication probably involves the formation of virus-like particles (VLPs), as in the case of retrovirids, metavirids and pseudovirids. Some belpaovirids carry an *env*-like gene [3], the function of which remains unknown.

## Genome

**Fig. 1.**
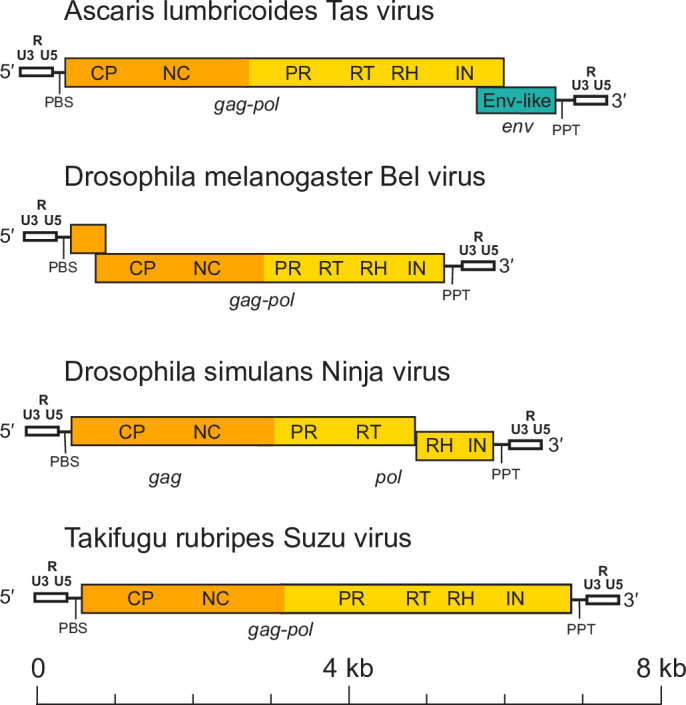
Full-length genome architectures of representative semotiviruses. Long terminal repeats, including the U3, R and U5 regions are coloured white, and the distinct *gag, pol* and *env* regions, are coloured orange, yellow and green, respectively. PBS, primer-binding site; PPT, polypurine tract; CP, capsid protein domain; NC, nucleocapsid protein domain; PR, protease; RT, reverse transcriptase; RH, ribonuclease H; INT, integrase; and Env-like, envelope-like protein.

Members of the family *Belpaoviridae* have a genomic organization typical of long terminal repeat (LTR) retrotransposons of the family *Metaviridae* [4], with one to three genes (*gag*, *pol* and *env*) being flanked by LTRs of 0.2–1.2 kb [5], and the whole genome being 4.2–10 kb. Downstream of the 5′-LTR is a non-coding region that corresponds to the first portion of the reverse-transcribed genome, followed by an 18 nt primer-binding site that is complementary to a specific region within the 3′-end of a host tRNA^Arg^ or a tRNA^Gly^. Upstream of the 3′-LTR is a polypurine tract of about 10 nt, which is responsible for priming the synthesis of the proviral DNA strand. The Gag and Pol polyproteins, encoding respectively the capsid and nucleocapsid domains, and the protease, reverse transcriptase (RT), ribonuclease H and integrase domains, can be encoded by either one continuous or two overlapping *gag* and *pol* genes (Table 1, Fig. 1) [6].

**Table 1.** Characteristics of members of the family *Belpaoviridae*

Example:	Ascaris lumbricoides Tas virus (Z29712), species *Ascaris lumbricoides Tas virus*, genus *Semotivirus*
Virion	Unknown
Genome	Linear single-stranded RNA of 4–10 kb
Replication	Replication by reverse-transcription primed by a host-encoded tRNA
Translation	Genomic RNA is translated into one or more polyproteins
Host range	Vertebrates, insects and nematodes
Taxonomy	Realm *Riboviria*, kingdom *Pararnavirae*, phylum *Artverviricota*, class *Revtraviricetes*, order *Ortervirales*; the genus *Semotivirus* includes >10 species

## Replication

Replication is poorly understood, but, given the similarity in both sequences and functional features, it is assumed to be similar to that of members of the family *Metaviridae* [4], in which RT mediates the conversion of a full transcript into a dsDNA that is integrated into the host genome by the integrase protein. The host RNA polymerase II then transcribes the integrated provirus to form new virus RNAs, which are capped and polyadenylated by the corresponding host enzymes. These new viral RNAs are exported to the cytoplasm of the host cell, where Gag and Pol are translated to form immature VLPs. The polyproteins are subsequently processed proteolytically by the viral protease, resulting in VLP maturation. RT then reverse-transcribes the new viral RNAs to dsDNA molecules, which are transported back to the nucleus, where they are inserted into new sites of the host cell genome [7].

## Taxonomy

Current taxonomy: www.ictv.global/taxonomy. *Semotivirus,* the only genus in the family, was previously placed in the family *Metaviridae*, but was removed due to its paraphyletic relationship with other genera of the family *Metaviridae*. Based on the currently known diversity of belpaovirids, additional species and genera are likely to be introduced into the family *Belpaoviridae* in the future. Within the order *Ortervirales*, members of the families *Belpaoviridae*, *Metaviridae* and *Retroviridae* have the same Pol domain order, whereas the integrase is either absent (*Caulimoviridae*) or located upstream of the protease domain (*Pseudoviridae*) [8].

## Resources

Full ICTV Report on the family *Belpaoviridae*: ictv.global/report/belpaoviridae.

GyDB: http://gydb.org

Repbase: https://www.girinst.org/repbase

RepetDB: http://urgi.versailles.inra.fr/repetdb

RexDB: http://repeatexplorer.org
